# *Nematostella vectensis*, an Emerging Model for Deciphering the Molecular and Cellular Mechanisms Underlying Whole-Body Regeneration

**DOI:** 10.3390/cells10102692

**Published:** 2021-10-08

**Authors:** Eric Röttinger

**Affiliations:** 1 Institute for Research on Cancer and Aging in Nice (IRCAN), CNRS, INSERM, Université Côte d’Azur, 06107 Nice, France; eric.rottinger@univ-cotedazur.fr; 2 Institut Fédératif de Recherche—Ressources Marines (IFR MARRES), Université Côte d’Azur, 06107 Nice, France

**Keywords:** whole-body regeneration, regeneration, functional genomics, stress response, *Nematostella vectensis*, cnidaria, anthozoa, sea anemone, marine organism

## Abstract

The capacity to regenerate lost or injured body parts is a widespread feature within metazoans and has intrigued scientists for centuries. One of the most extreme types of regeneration is the so-called whole body regenerative capacity, which enables regeneration of fully functional organisms from isolated body parts. While not exclusive to this habitat, whole body regeneration is widespread in aquatic/marine invertebrates. Over the past decade, new whole-body research models have emerged that complement the historical models *Hydra* and planarians. Among these, the sea anemone *Nematostella vectensis* has attracted increasing interest in regard to deciphering the cellular and molecular mechanisms underlying the whole-body regeneration process. This manuscript will present an overview of the biological features of this anthozoan cnidarian as well as the available tools and resources that have been developed by the scientific community studying *Nematostella*. I will further review our current understanding of the cellular and molecular mechanisms underlying whole-body regeneration in this marine organism, with emphasis on how comparing embryonic development and regeneration in the same organism provides insight into regeneration specific elements.

## 1. Introduction

Regeneration is the capacity of tissues, organs, or even entire organisms to regrow lost or injured body parts and, therefore, maintain function and extend longevity. Regeneration is widespread, yet highly variable among animals and has intrigued scientists and philosophers for centuries [1], as some organisms can and others, such as humans, cannot (or can, but to a lesser extent) [2]. This particular developmental trajectory involves various cellular processes, such as reprogramming, de-differentiation or trans-differentiation that can operate in different tissues of the same animal [3].

During the multi-factorial process of aging, certain tissues, organs, or body parts lose their capacities to regenerate, leading to age-related diseases, like organ failures. Interestingly, in some organisms, whole-body regeneration (WBR) is associated with extended longevity [4,5]. In fact, these latter animals are able to reform fully functional organisms from small body fragments within days after injury and display no naturally occurring age-related diseases. 

The aquatic/marine biodiversity harbors a large array of invertebrates that possess these fascinating WBR capacities that are absent or limited in most established invertebrate or vertebrate research models, such as *Drosophila*, *Caenorhabditis elegans,* or mice. While *Hydra* (hydrozoan cnidarian) and planarians (Platyhelminthes) are the historical WBR research models [6,7], over the past decades, additional and complementary WBR research models spanning the metazoan tree of life have (re-)emerged [2]. Scientists working on the latter have benefitted from the development of new sequencing, imaging, and functional genomics technologies [2,8,9,10,11,12,13,14,15,16]. 

Among these emerging WBR models, the anthozoan cnidarian *Nematostella vectensis* has attracted increasing interest, in regard to deciphering the cellular and molecular mechanisms underlying the reformation of after injury. The reasons are multiple, and include its phylogenetic position [17], a conserved gene content and genome architecture compared to vertebrates [18], its relatively simple morphology, the handiness to rear them under laboratory conditions as well as a wealth of resources, and tools, such as CRISPR/Cas9-mediated genome editing [19]. Another interesting aspect of this model is that it is suitable to compare the molecular mechanisms underlying embryonic development and regeneration within the same organism in order to highlight similarities as well as regeneration specific features [19,20,21]. 

In this review, I will provide a general presentation of this emerging research model, the available resources, techniques and tools for functional genomics, as well as an up-to-date view of the tissular, cellular, and molecular events that trigger and drive WBR in *Nematostella*.

## 2. Phylogeny, Morphology, Reproduction, and Homeostatic Plasticity of *Nematostella vectensis*

**Figure 1:**
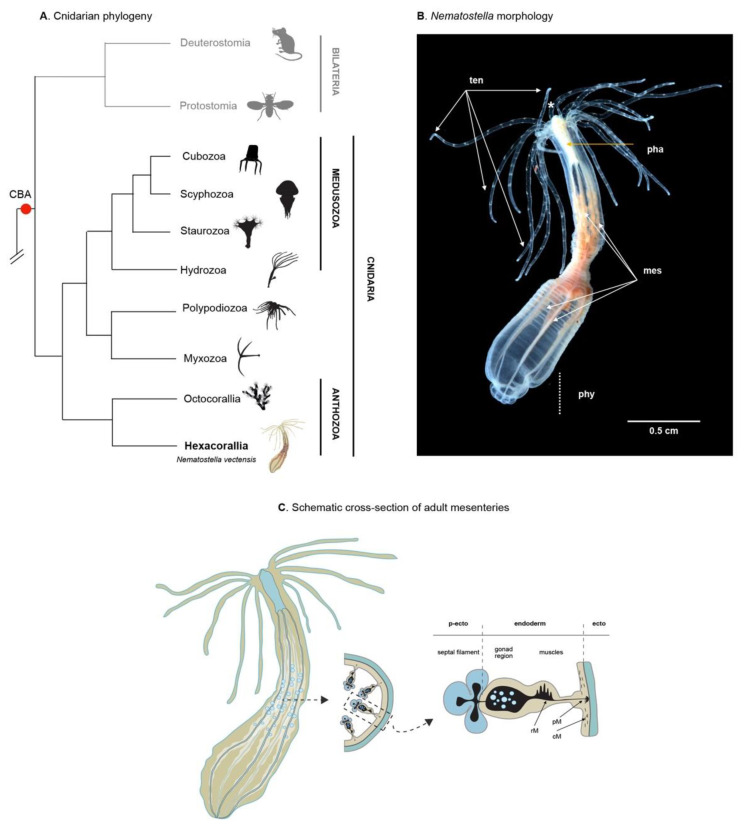
(**A**) Simplified metazoan phylogeny, emphasizing the position of cnidaria as a sister group to bilaterians as well as the cnidarian phylogeny highlighting the relationships between the major groups. CBA (cnidarian-bilaterian ancestor). Data based on [89,90]. (**B**) Adult *Nematostella* polyp. (*) mouth, (ten) tentacles, (pha) pharynx, (mes) mesenteries (phy) physa. Figure modified from [91,92]. (**C**) Schematic cross section of a complete/adult mesenteries at the gonadal section (adapted from [83,93]). (p-ecto) pharyngeal ectoderm, (ecto) ectoderm, (rM) retractor muscle, (pM) parietal muscle, (cM) circular muscle.

Cnidarians (jellyfish, *Hydra*, corals, gorgonians, sea anemones) are strictly aquatic animals and considered the “sister taxon” to bilaterians (Figure 1A). As such, they hold an important phylogenic position for studying the evolution of developmental processes and the emergence of biological novelties [22]. Cnidarians are diploblastic animals formed by two germ layers, the ectoderm and the endoderm (also called endomesoderm, gastrodermis, or entoderm), lacking a well-defined and segregated mesoderm [23,24]. 

During the early phase of the evo–devo field [25], the scientific community was in search for cnidarian models (i) that complement the historical hydrozoan cnidarian, *Hydra* [26,27], and (ii) with access to embryonic material and the development of functional genomic tools. In this context, in the 1990s, Hand and Uhlinger published a protocol for rearing, induction of spawning, and completing the entire life cycle under laboratory conditions of the anthozoan cnidarian sea anemone *Nematostella vectensis* [28]. Since then, scientists have adopted this new cnidarian research model for gaining insight into the evolution of genomes as well as molecular mechanisms underlying embryonic development, i.e., germ layer formation, axial patterning, or cell-type specification, leading to a wealth of publications (reviewed in [19,29,30], resources, and tools (Table 1).

Adult *Nematostella* polyps are translucent and approximatively 1–3 cm in size (Figure 1B). In the wild, they are found in estuarine environments, buried in the sand/mud with only the head region/tentacles extruding from the surface to catch food from the water column. Once paralyzed by toxins present in the cnidocytes (stinging cells present throughout the body and very abundant in the tentacles), the prey is brought to the mouth, ingested, and then digested within the body cavity via secreted enzymes and continuous peristaltic movements. The digestive enzymes are secreted by cells located in the mesenteries, internal structures that also serve as lipid storage and that harbor the gonads [83]. Unlike most sea anemones that have a foot to attach to the substrate, the aboral extremity of *Nematostella* is a non-adhesive structure called the physa that together with muscle-driven peristaltic movements facilitates burrowing in the mud. 

A recent study revealed that *Nematostella* is composed of at least eight cell classes (myoepithelial cells, neuronal cells, gland cells, digestive filaments, cnidocytes, ectodermal and endodermal epithelial cells, and progenitor cells), each of which comprises different class-specific subtypes totaling more than 100 cell states [55]. As in all diploblastic animals, all body structures of *Nematostella*, including the mesenteries, are composed of only two germ layers, ectoderm (also called epidermis) and endoderm (also called endomesoderm or gastrodermis). These two epithelial layers are separated by the mesoglea, a largely acellular matrix containing a sparse number of amoeboid cells [86]. 

The body wall ectoderm serves as a protective barrier between the animal and its environment and is made up mostly of ectodermal epithelial cells, neurons, gland cells and cnidocytes. The body wall endoderm harbors myoepithelial cells that form the circular muscles and neurons. The endodermal component of the eight mesenteries (tissue infoldings) is formed during larval development [36,37,74,83]. In polyps, this mesenterial endoderm contains myoepithelial cells that form the retractor and parietal muscles, the gonad, as well as cells involved in nutrient absorption and storage (Figure 1C) [83,87]. Fate mapping experiments using transgenic grafting experiments, have shown the ectodermal origin of the pharynx and the septal filaments. The septal filaments that correspond to the mesenterial ectoderm are primarily composed of epithelial cells, cnidocytes, and gland cells that secrete the digestive enzymes (Figure 1C) [83].

*Nematostella* possesses a diffuse nerve net formed by a variety of interconnected neuronal sub-types (e.g., sensory or motor-sensory cells, glandular cells, cnidocytes) that originate from both the ectoderm and the endomesoderm [49,81]. Although primarily diffuse, the nervous system is nonetheless organized in visible structures such as neuronal condensations along the mesenteries, or nerve rings around the mouth and pharynx [76,88]. 

**Figure 2:**
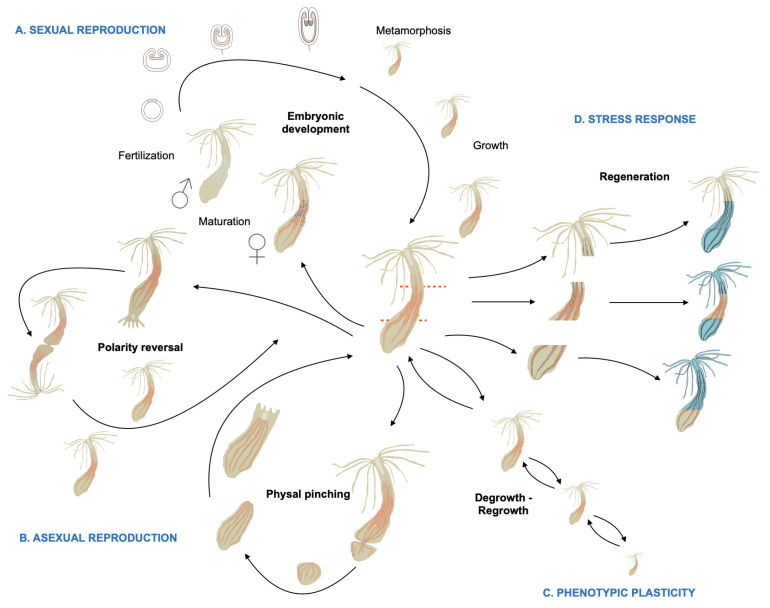
Schematic representation of the various developmental trajectories and the phenotypic plasticity of *Nematostella*: Embryonic development, polarity reversal, physal pinching, degrowth and regrowth, whole-body regeneration (modified from [92]).

While there is no sexual dimorphism in *Nematostella,* this sea anemone is gonochoric (sexes are separate) and able to reproduce sexually. In addition, *Nematostella* can also reproduce asexually via two specific types of budding (polarity reversal and physal pinching, see below), undergo whole body regeneration upon injury and is able to cope phenotypically with environmental variations, in particular in response to food availability (Figure 2A). Upon stimulation, gametes are released into the water column where the sperm fertilizes the mature egg. Under laboratory conditions, one can select females by sorting out the polyps that release egg masses. In addition, spawning can be induced via a light and temperature stimulus and fertilization can be precisely controlled by spawning sexes separately [28,31,33]. After fertilization, the egg begins to cleave, develop into a blastula that gastrulates at the animal pole [38], resulting in a swimming planula larva that gradually metamorphoses after a few days to give rise to a juvenile *Nematostella* polyp [94]. Metamorphosis in *Nematostella* consists in reducing/stopping its natatory behavior, loss of its apical tuft, formation of the tentacle crown, and initiating the final steps of pharynx and mouth formation [28,39]. Primary juvenile polyps possess four tentacles and two complete mesenteries (endodermal + ectodermal component). Both progressively increase in number during the growth of the polyp resulting in at least 16 tentacles and 8 mesenteries when the animals are sexually mature. The growth of the polyp to reach adult characteristics is nutrient- and temperature-dependent. When those conditions are optimized, the entire life cycle can be completed in about 8 weeks (Carvalho, Amiel, and Röttinger, unpublished).

In addition to embryonic development, asexual developmental trajectories, such as polarity reversal and physal pinching (Figure 2B), have been reported in *Nematostella* [28,95,96]. During polarity reversal, a second oral crown forms at the aboral site where the physa is located. This process that can take up to several months and leads to the development of two fused individuals, that feed independently and eventually separate to form two clonal polyps [28,95,96]. Physal pinching also leads to the formation of two clonal polyps; however, the starting point is the physa. A constriction appears at the aboral-most region of the body column that leads to the separation of this body part. The isolated physa contains portions of one or more parental mesenteries; mouth, pharynx and tentacles are absent. After separation, the number of complete mesenteries grows back to eight, and the pharynx, mouth, and tentacles form, enabling the pinched physa to begin feeding 3–5 days after separation [28,95,96]. Unfortunately, very little is known about the stimuli that trigger one or the other type of asexual reproduction. 

The few studies that investigated this phenomenon suggest that this might be linked to food availability and the feeding regime [95]. However, pinching was also observed under starved conditions. Different populations fed under equal conditions lead to various number or clones. Thus, genetic or epigenetic factors might be involved in this process as well [96]. In regards to feeding regime, it is important to note that the phenotypic plasticity of *Nematostella* enables this sea anemone to adapt to the food availability not only by producing new clones when food is abundant, but also by reducing its mitotic activity [97] and de-growing in size when food is absent (Röttinger, unpublished). As soon as food becomes available, mitotic activity is reactivated and the regrowth of the polyp starts over again (Figure 2C). A recent study, using a transgenic reporter line (*NvLWamide-likemCherry*) that labels neuronal sub-types has shown that, in response to the feeding/starvation regime, the body not only alters its body size, but also alters the numbers of neurons to reflect the resulting size without affecting the polyp behavior [98].

Finally, upon physical stresses, such as injury or bisection, *Nematostella’s* whole-body regenerative capacity (Figure 2D) enables it to regrow lost/missing body parts within days, even from small, isolated fragments [96,99]. While the developmental and genomics aspects of *Nematostella* have been the subjects of a fair number of studies (444 publications according to PubMed as of April 2021) since its initial developmental description in 1992 (Hand), whole-body regeneration in *Nematostella* has been investigated in less than 15 studies since 2006 when this topic had reemerged. The current knowledge of the tissular, cellular, and molecular mechanism underlying whole-body regeneration from these studies will be covered in detail in the sections below.

## 3. Available Resources, Techniques, and Tools to Study *Nematostella vectensis*

Table 1 summarizes current resources, techniques, and tools available for studying *Nematostella*. The first non-bilaterian genome that was sequenced and published was the one from *Nematostella* in 2007 [18] (Table 1). Unexpectedly, this genome revealed an astonishing conservation with vertebrates (i.e., human frog and pufferfish genomes) in terms of gene content, gene synteny, as well as intron/exon organization of orthologous genes [18]. A genome-wide map of gene regulatory elements revealed that the epigenetic regulation is also more conserved with bilaterians than initially expected [56]. In fact, this study showed that enhancers of developmental genes in *Nematostella* share the same/similar combination of histone modifications as the ones found in enhancers in bilaterians. However, posttranscriptional regulation appears to be different from bilaterians but similar to plants [100]. An updated and improved version of the genome using long-read sequencing approaches was recently released [48] (Table 1). 

Since the initial genome sequencing, a wealth of studies have assessed spatial gene expression of developmental/axial patterning genes as well as members of evolutionarily conserved signaling pathways during embryonic and larval development [50,51,57,58,65,101] (and references in Table 1). More recently, a variety of -omics data were produced for *Nematostella* including RNA-seq, ChIP-seq, as well as ATAC-seq from either whole organisms or single cells [20,55,56], as well as references in Table 1. At the same time, the scientific community has developed a variety of functional genomics tools that range from mRNA or morpholino injection to meganuclease-based transgenesis or CRISPR/Cas9-induced knock-in (KI) and knock-out (KO) approaches [39,40,41,68,69,70,74] and references in Table 1. The latter approaches have led to the development of a growing number of KO lines that are primarily useful to study the roles of the genes of interest during embryonic development, as well as genetic reporter lines useful for a variety of global or cell-type specific analysis [68,69,70,74,84], as well as references in Table 1.

## 4. The Morphological, Cellular, and Molecular Basis of Regeneration in *Nematostella*

Following natural (predation) or artificially induced (bisection) injury that can lead to the loss of body parts, *Nematostella* is able to regrow the latter within a few days. This regenerative capacity was initially described by Hand and Uhlinger, and only periodically investigated since then [96,99]. The adaptation and development of new techniques/tools to investigate regeneration in *Nematostella*, have recently provided new insights into this process at the morphological, cellular, and molecular levels.

### 4.1. Morphological and Tissular Dynamics

The original description of regeneration in *Nematostella* referred to the reformation of missing body parts following physal pinching (also named posterior budding, [95]). This physiological type of regeneration appears to be variable in regard to the timing between the detachment and first ingestion of food that can vary between 3 days and several weeks [95]. The different types of asexual reproduction, i.e., physal pinching and injury-induced regeneration, were investigated a decade later, showing that, independent of the location of the bisection site, adult polyps are able to regrow missing body parts within weeks. This study emphasized on the difference between i) endogenously triggered constriction at the pinching site, and ii) exogenously triggered constriction/wound healing at the injury site, which appear to be two distinct onsets of the regenerative process. In addition, the authors described irregular/incomplete regeneration leading to the formation of ectopic heads that could be associated to the dissection plane [96].

An in-depth investigation of the ultrastructure of the mesoglea (the extracellular matrix separating the epidermis and endodermis) revealed modifications of the basal lamina and fibril depositions at early phases of the regeneration process that are completely recovered after 5 days following amputation [86]. To develop a universal protocol that describes the morphological dynamics underlying regeneration in *Nematostella*, Bossert and colleagues described a staging system following supra-physal bisection in adult polyps [102]. In addition to highlighting discernable morphological features for the progression of regeneration, the authors also show that wound healing is temperature independent, while subsequent progression varies according to the temperature that the regenerating polyps are maintained at. The described stages correspond to morphological events that likely also happen during physal pinching making this study a very interesting basis for comparing asexual propagation trajectories once the induction of budding can be controlled. However, the size and opacity of adult tissues make them more difficult to analyze using microscopic imaging approaches. Furthermore, the majority of regeneration studies in *Nematostella* have focused on mid-body or sub-pharyngeal amputations in either adults or juveniles [13,97,103]. 

**Figure 3:**
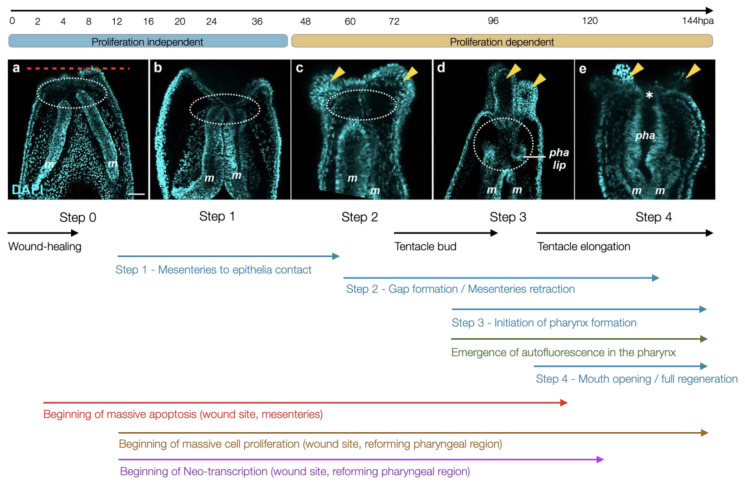
Overview of the morphological hallmarks characterizing whole-body regeneration in *Nematostella*. Confocal images of DAPI (nuclei in cyan) stained regenerating oral regions of juvenile *Nematostella* polyps (a–e) represent the characteristic phenotype of a given regeneration step. The panel spans 144 hours of regeneration and indicates the proliferation independent (steps 0, 1) and dependent (steps 2–4) steps of the process. The arrows below indicate the beginning of measurable tissular, cellular, and molecular events. The red dashed line indicates the amputation plane and the yellow arrow heads highlight tentacle buds/elongating tentacles; (m) mesenteries, (pha lip) pharyngeal lip, (pha) pharynx, (*) mouth opening (modified from [21,77,99]).

To set a common groundwork for comparing regeneration studies in *Nematostella*, Amiel and colleagues compared oral regeneration in juveniles and adults revealing that the regenerative capacity and the timing of regeneration are age-independent following sub-pharyngeal amputation [77]. A detailed analysis of the morphological events that are triggered by the amputation have highlighted four characteristic stages of regeneration in *Nematostella* (Figure 3). Following injury (step 0), the wound heals (step 0.5), and the mesenteries contact one another as well as the amputation site (step 1). Simultaneously with the moment that the tentacle buds appear, a gap between the oral part of the mesenteries and the amputation site becomes visible (step 2), which corresponds to a highly proliferative region where the pharyngeal lip develops (step 3) followed by the reformation of the pharynx and the re-opening of the mouth (step 4). This study has highlighted that the early steps (0, 1) are proliferation independent, while later steps (2–4) require mitotic activity. The authors also developed in vivo assays to assess (i) completion of wound healing that is terminated by 6 h post amputation (hpa)); (ii) the reformation of the pharyngeal lip/pharynx that is initiated at 72 hpa and visible via the reappearance of autofluorescence in that region; as well as (iii) the contribution of pre-existing tissue to the reformation of lost body parts. The latter has revealed that the remaining oral part of the mesenteries contribute in large part to the reformation of the pharynx, while the body wall epithelium of the amputation site is at the origin of the mouth opening and the tentacles [77].

The oral regeneration process occurs without the intake of major nutrients as the mouth is absent until day 7. Thus, regeneration results in polyps that are smaller in size than the pre-amputated animal [98]. In line with the results observed in de-grown animals mentioned above, the number of LWamide positive neurons is also reduced in these regenerates [98]. Interestingly, different LWamide neuronal subtypes displayed subtype-specific responses during regeneration. In fact, this study shows that there are at least three distinct neuronal responses after the onset of regeneration; (i) continuous increase of pharyngeal and tentacular neurons; (ii) invariable tripolar neurons; and (iii) longitudinal neurons that decrease during the first 24 hpa, then increase to recover numbers present in the 0 hpa fragments [98].

Although *Hydra* and planarians are considered to regenerate all body parts, certain regions, such as the tentacles or the foot in *Hydra* as well as the pharynx in planarians, are incapable of regenerating when isolated. This is due to fact that those regions are highly differentiated and lack adult stem cells, namely i-cells and neoblast in *Hydra* and planarians, respectively [104,105]. Adults stem cells have yet to be described in anthozoan cnidarians (sea anemones and corals), including *Nematostella*. To initiate a study determining the existence of adult stem cells in *Nematostella*, Amiel and colleagues have assessed the limits of the regenerative capacity in this anthozoan model [99]. Surprisingly, this study has revealed that supra-physal amputation leads to the regeneration of lost body parts in an age-dependent manner, i.e., isolated adult physa regenerate, while isolated juvenile physa cannot regenerate. The lack of properly formed mesenteries in the physa of juveniles indicate that these internal structures are required for regeneration to occur. In fact, when the mesenteries are experimentally removed from the body column the remaining tissue (isolated body wall epithelia) fails to regenerate lost body parts. When the mesenteries are grafted back into the isolated body wall epithelia, proliferation is induced, and the regeneration process reactivated [99]. This is in line with the staging system that described the contact between the mesenteries and the amputation site at the earliest step of regeneration (step 1) following amputation and wound healing [77]. The molecular signal emitted by the mesenteries and received by the body wall epithelia at the amputation site is yet to be identified and characterized.

### 4.2. Cellular Dynamics Underlying Nematostella Regeneration

Similar to all metazoans that possess regenerative capacities [106], cellular proliferation is also crucial for regenerating lost body parts in *Nematostella.* In fact, while homeostatic proliferation increases after feeding and reduces over time in starving animals, the amputation stress in the latter triggers a regeneration-specific proliferative response independent of the starvation state [77,97]. Prior to a burst of cell proliferation at 18–24 hpa [77,97], mitotic activity is detected at the amputation site as soon as 12 hpa in juveniles and adults [35,97]. Furthermore, blocking cell proliferation using Hydroxyurea (HU) completely arrests regeneration [77,97], and this at a very early stage, i.e., step 0.5 [77,97]. Inhibition of regeneration by HU treatments is similar in juveniles and adults [77,97], further confirming that regeneration in *Nematostella* is age-independent following sub-pharyngeal amputation [77]. 

**Figure 4:**
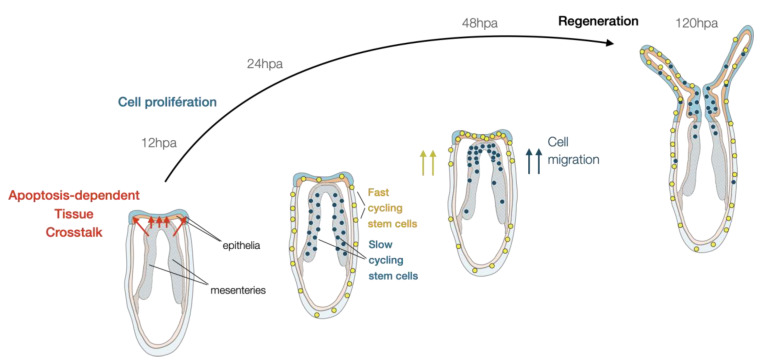
Current model of the cellular dynamics underlying whole body regeneration in *Nematostella*. Following sub-pharyngeal amputation, the tissue crosstalk between the mesenteries and the body wall epithelia at the amputation site triggers a regenerative response via the activation of two potential stem cell population (yellow and blue) that migrate towards the amputation site where they start dividing and participate in the reformation of lost body parts (modified from [21,77,99].

A study in *Hydra* has revealed that injury-induced apoptosis is crucial for the activation of cell proliferation in neighboring i-cells [107], a process that has also been described in other regeneration models [108]. In a similar manner, apoptosis in *Nematostella* is activated shortly after puncture [13] or amputation [21] in cells close to the injury site. Apoptotic cells remain at the amputation site until at least 12 hpa when cellular proliferation becomes visible in the same region. At later regeneration time points, apoptotic cells are detected more broadly in the body with an increased occurrence in the mesenteries [21]. Inhibition of apoptosis using a pan-caspase inhibitor inhibits the initiation of cell proliferation and blocks regeneration at a very early stage, just after wound-healing (step 0.5). In fact, when apoptosis is blocked, the contact between the mesenteries and the epithelia of the amputation site does not occur [21]; thus, preventing the tissue crosstalk required for initiating a regenerative response [99] (Figure 4). 

Irradiation in *Nematostella* affects homeostatic proliferating cells and blocks regeneration at an early step [99]. This observation is consistent with what has been described in other whole-body regeneration models such as planarians [6]. However, the study in *Nematostella* has also revealed that a population of small cells with compact DNA, located at the tip of the mesenteries, escaped irradiation, and re-entered mitosis 48 after amputation. This observation suggested the presence of slow-cycling or quiescent cells similar to adult stem cells in bilaterians. Combining a series of EdU pulse and chase with grafting and amputation experiments, this study identified two populations of cells that i) are fast or slow cycling/quiescent, predominantly present in the body wall epithelia and the mesenteries, respectively; ii) are stimulated upon injury; and iii) migrate towards the amputation site and participate in the reformation of lost body parts [99] (Figure 4). While those populations of cells share certain characteristics of adult stem cells, their capacity to self-renew, their molecular identity, and potency remain unknown. Interestingly, a recent scRNA-seq study performed on adult *Nematostella* polyps has revealed a set of genes (e.g., *piwi*, *myc*, *nanos*) expressed in specific cell populations and that define so-called “progenitor” cell states [55]. These genes have been described as germ/stem cell markers in other marine invertebrates [109,110,111,112,113,114,115,116,117], suggesting that they might be expressed in either one or both of the above-mentioned potential stem cell populations in *Nematostella*.

### 4.3. Molecular Wound-Healing Response and Patterning during Nematostella Regeneration

To investigate the roles of MEK/ERK, Notch, and TGFβ signaling on the wound-healing response and regeneration, Dubuc and colleagues performed a series of experiments with pharmacological inhibition of these signaling pathways. Wound-healing was assessed following puncture in the body wall epithelia and regeneration following sub-pharyngeal amputation. They showed that i) inhibiting TGFβ has no effect either on wound-healing or regeneration; ii) the inhibition of Notch signaling only blocks regeneration, while iii) inhibiting MEK/ERK signaling prevents wound-healing following puncturing the body wall epithelium as well as oral regeneration [13]. A recent study has confirmed the role of this pathway in regeneration that is blocked at a very early phase (step 0.5) after the inhibition of MEK/ERK signaling. However, this study has also shown that wound-healing following sub-pharyngeal amputation is not blocked but significantly delayed [21]. Although important to mention, this difference might simply be due to the different types of injuries (puncture vs bisection) in those two studies. 

Interestingly, unlike in *Hydra,* where MAPK signaling activates apoptosis [118], inhibition of MEK/ERK does not affect apoptosis in *Nematostella* following puncture-induced injury [13]. Whether this is also the case following sub-pharyngeal amputation is yet to be determined. Furthermore, this study has revealed a set of MEK/ERK downstream targets during wound-healing including matrix metalloproteinases that might be responsible for the activation of growth factors involved in later stages of the wound-healing process and potentially at the onset of regeneration [13]. Another study, discussed below in the context of comparing embryonic development and regeneration at the Gene Regulatory Network (GRN) level, has determined MEK/ERK downstream targets during the onset of regeneration [21].

The canonical Wnt (cWnt) pathway has been shown to be involved in regeneration and patterning the reformation of lost body parts in a large variety of organisms [119] including the whole-body regeneration models *Hydra* and planarians [120,121,122,123,124]. In a similar manner, cWnt in *Nematostella* is involved in specifying oral fates during regeneration as hyperactivation of this signaling pathway is sufficient to induce the formation of ectopic oral structures [78]. Strikingly, this global role appears to be conserved between embryonic development and regeneration [40,51,78]. 

While most of the studies in *Nematostella* focused on regenerating the lost oral body parts, a study in 2016 compared the transcriptional dynamics following the amputation of oral *vs* aboral body parts [125]. The authors performed a transcriptomic time course spanning five time points over the course of five days [125]. While the genes dynamically expressed during early time points appear closely related between the oral and the aboral regenerating regions, at later time points becomes more divergent in each fragment. The latter seems to reflect the onset of differentiation of oral or physal fates. While the similar transcriptional responses at early time points likely reflects similarity of wound-healing mechanisms [125]. The characterization of the isolated body part specific gene response revealed that several GO groups are enriched during oral regeneration, including those that involve cWnt signaling, chitin metabolic process, as well as microtubule and cilia-associated genes. The authors propose that the latter group could contain novel players responsible for the induction of polarization of the regenerating body parts in *Nematostella* or more broadly in whole body regenerating organisms [125]. 

### 4.4. Regeneration-Specific Genes and Gene Modules

One of the interesting features of *Nematostella* is that one can access and study embryonic development as well as whole body regeneration. Thus, unlike *Hydra* and planarians in which embryonic development is more difficult to study, *Nematostella* is well suited to investigate the historical question about the relationship between embryonic development and regeneration, two developmental trajectories that lead to a fully functional organism in less than a week. The available functional genomics tools as well as the blueprints of embryonic gene regulatory networks [51,65,76], makes this sea anemone particularly interesting to compare the gene regulatory networks (GRN) underlying embryonic development and regeneration to decipher shared as well as regeneration-specific gene modules and genes [19]. The first study that investigated the molecular relationship between embryonic development and regeneration in *Nematostella* was carried out by Burton and colleagues [103]. In this study, the authors compared *in situ* hybridization gene expression patterns of the transcription factors *otxC*, *anthox1*, *anthox6*, *anthox8,* and *foxA*. While some of these genes displayed expression during regeneration that may resemble the one of embryonic development (ex. *foxA*), other genes were not detected or expressed in a different manner (ex. *otxC*), suggesting a partial reactivation of developmental genes during regeneration [103]. 

**Figure 5:**
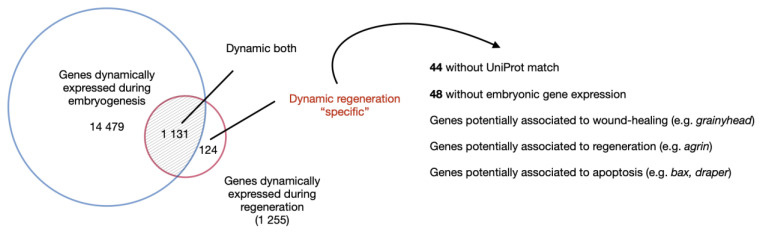
Regeneration is a modest transcriptomic event compared to embryonic development. Comparison of the transcriptomic time series during embryonic development and regeneration revealed that regeneration is reactivating a modest number of genes transcriptionally dynamic during embryonic development. Interestingly, this comparative approach also enables the identification of genes with a regeneration-specific expression dynamics (adapted from [21,35,56]).

Warner and colleagues performed an extensive transcriptomic analysis during oral regeneration to investigate this question more globally at the transcriptomic and gene regulatory network levels. The authors also complemented existing transcriptomic data from embryonic and larval development, metamorphosis, and post-metamorphic growth [20,21]. A global comparison of these data revealed that transcriptional changes caused by regeneration are modest compared to those observed during embryogenesis. This study also identified a set of genes with regeneration-specific expression dynamics. Some of these genes were associated with apoptosis (e.g., *bax*, Figure 5, [21]). Genes associated to apoptosis were also detected in a co-expression module whose gene content and expression pattern was coregulated during regeneration, but not during embryonic development. Inhibition of apoptosis following fertilization did not affect embryonic development or metamorphosis. However, as indicated above, inhibition of apoptosis following sub-pharyngeal amputation blocked regeneration just after the wound has healed. Those results further support the idea that apoptosis in *Nematostella* is a regeneration-specific process [21]. The precise mode of activation of apoptosis and the mechanism by which apoptotic cells control the tissue crosstalk and the onset of regeneration remains to be elucidated. 

From a GRN point of view, this comparative work suggested that regeneration deploys a novel GRN logic to activate the regeneration process. Thus, the authors tested this hypothesis by comparing the GRN modules mediated by MEK/ERK signaling during embryonic development [65,76] with the ones deployed during regeneration [21]. Molecular analysis following inhibition of MEK/ERK signaling at the onset of regeneration revealed that this pathway controls only a subset of embryonic MEK/ERK downstream targets. Furthermore, MEK/ERK signaling also integrates additional genes in its regeneration GRN that were not part of the embryonic GRN. In particular, downstream targets controlled by cWNT signaling during embryonic development as well as genes that belong to the above-mentioned set of “regeneration-specific” genes [21]. While this work lays down the framework for a regeneration GRN in *Nematostella*, the molecular elements, i.e., enhancers and transcription factors responsible for such a regeneration-specific network, remain unknown. 

## 5. Conclusions and Future Directions

The development of novel and original research models, in particular marine organisms with extraordinary properties, such as whole-body regeneration and an extended lifespan, are crucial to increase our basic understanding underlying these features. The anthozoan sea anemone, *Nematostella vectensis*, is such an example. Thanks to (i) its “simple” morphology; (ii) the vast functional toolkit developed by the community working on this model; (iii) the shared genomic organization and gene content with vertebrates; as well as (iv) the accessibility to study embryonic development and whole body regeneration, could help us to understand the cellular and molecular mechanisms underlying this biological feature, which is largely absent in “classical” research models, such as the fruit fly, nematodes, or mammals. Gaining insight into these mechanisms, combined with a comparative approach, including regenerating as well as non-regenerating metazoans, may also yield an increased understanding of why certain animals regenerate while others do not, or have reduced regenerative capacity.

Although studies investigating the whole-body regenerative capacity in *Nematostella* are sparse, the above-mentioned studies provide first hints into the tissular, cellular, and molecular dynamics underlying this process, and pave the way for future work exploiting this exciting research model. Not only are apoptosis and cell proliferation crucial for inducing a regenerative response in *Nematostella*, but apoptosis is also a regeneration-specific event that is crucial to enable a tissue crosstalk between the mesenteries and the amputation site that triggers a cellular response involving potential stem cell populations [21]. The regeneration-specific role of apoptosis was revealed by taking advantage of the capacity to compare embryonic development and whole-body regeneration in *Nematostella* at the molecular levels. Comparing oral vs. aboral regeneration, or embryonic development vs. regeneration in *Nematostella* provides a specific entry point into the molecular aspects of regeneration in this model. The latter approach provides a first glimpse into how the embryonic GRN is rewired and interconnected with regeneration specific components in order to quickly regenerate lost body parts. Other whole-body regeneration models, such as the acoel *Hofstenia miamia* [10], have emerged in recent years to decipher the GRNs underlying this process, enabling the community to compare the data between species, understand the shared regulatory mechanisms by which regeneration-specific gene expression occurs, and potentially identify shared core modules conserved in whole-body regeneration models. 

Much remains to be studied in *Nematostella* to gain a broader understanding, not only on the whole-body regeneration capacity and stem cell biology of this sea anemone, but also on the general phenotypic plasticity and capacity to respond to environmental variations and stresses (e.g., starving, salinity variations) and how these mechanisms are linked to its potentially extended lifespan and longevity. Extensive tools exist to study embryonic and larval development, and the *Nematostella* community is actively developing functional tools, such as biosensors or conditional KO approaches, which are required to perturb and study gene functions in juveniles and adults during homeostasis and regeneration. Unconventional research models, in particular marine organisms with fascinating biological features, enable us not only to tackle historical questions from a different angle, but also fuel our research with original biological questions that inform us about the fundamentals of metazoan biology and evolution.

## Figures and Tables

**Table 1:** Overview of techniques, resources, tools, and lines available for functional genomics studies in *Nematostella*. Updated and extended from [19].

**Culture and Experimentation**	
Rearing and spawning	[28,31,32]
Inducing and assessing regeneration	[33,34,35]
Microinjection and micromanipulation	[36,37,38,39,40,41,42,43,44,45,46,47]
**Genome, Resources, and Protocols for -Omics Analyses**	
Annotated genome—V1.0	[18] https://mycocosm.jgi.doe.gov/Nemve1/Nemve1.home.html (accessed on 5 October 2021)
Annotated genome—V2.0	[48] https://genomes.stowers.org/starletseaanemone (accessed on 5 October 2021)
Microarrays	[49,50,51,52]
RNA-seq/transcriptomes	[20,53,54] http://nvertx.kahikai.org (accessed on 5 October 2021)
scRNA-seq	[55]
ATAC-seq	[55]
ChIP-seq	[56]
**Spatio–Temporal Gene Expression Analysis**	
mRNA in situ	[57,58,59,60,61,62,63,64]
Immunohistochemistry	[36,37,39,40,46,65,66,67]
Transgenic reporter	[68,69,70,71,72,73]
**Tools to Study Gene Function**	
mRNA over-expression	[40,47,49,51,65]
Morpholino	[36,39,41,46,47,49,51,63,65]
Short hairpin RNA	[74,75]
TALEN/Fok1, CRISPR/Cas9	[69]
Heat-shock inducible promoters	[69]
Pharmaceutical treatments	[21,39,51,71,76,77,78,79,80]
**Available Stable Reporter Lines**	
NvMyHC::mCherry	[68]
NvElav::mOrange	[81]
NvElav::Cerulean	[82]
NvLWamide:mCherry	[73]
NvEf1a::mOrange-CAAX	[83]
NvFoxQ2d::mOrange-CAAX	[82]
NvNcol-3::memOrange2	[84]
NvAnthox8::Gfp	[74]
NvPou4::mCherry	[70]
NvTBP::mCherry	[85]
**Available Stable KO Lines**	
NvAnthox1a^−/−^	[74]
NvAnthox6^−/−^	[74]
NvAnthox8a^−/−^	[74]
NvFgfrB^+/−^	[71]
NvPou4^+/−^	[70]
